# Spatial and Temporal Analysis of the Stomach and Small-Intestinal Microbiota in Fasted Healthy Humans

**DOI:** 10.1128/mSphere.00126-19

**Published:** 2019-03-13

**Authors:** Anna M. Seekatz, Matthew K. Schnizlein, Mark J. Koenigsknecht, Jason R. Baker, William L. Hasler, Barry E. Bleske, Vincent B. Young, Duxin Sun

**Affiliations:** a Department of Internal Medicine, Division of Infectious Disease, University of Michigan, Ann Arbor, Michigan, USA; b Department of Microbiology and Immunology, University of Michigan, Ann Arbor, Michigan, USA; c Department of Pharmaceutical Sciences, College of Pharmacy, University of Michigan, Ann Arbor, Michigan, USA; d Department of Internal Medicine, Division of Gastroenterology and Hepatology, University of Michigan, Ann Arbor, Michigan, USA; e Department of Pharmacy Practice and Administrative Sciences, University of New Mexico, Albuquerque, New Mexico, USA; University of Wisconsin—Madison

**Keywords:** mesalamine, microbiota, pH, small intestine, stomach

## Abstract

Although the microbiota in the proximal gastrointestinal (GI) tract have been implicated in health and disease, much about these microbes remains understudied compared to those in the distal GI tract. This study characterized the microbiota across multiple proximal GI sites over time in healthy individuals. As part of a study of the pharmacokinetics of oral mesalamine administration, healthy, fasted volunteers (*n* = 8; 10 observation periods total) were orally intubated with a four-lumen catheter with multiple aspiration ports. Samples were taken from stomach, duodenal, and multiple jejunal sites, sampling hourly (≤7 h) to measure mesalamine (administered at *t* = 0), pH, and 16S rRNA gene-based composition. We observed a predominance of Firmicutes across proximal GI sites, with significant variation compared to stool. The microbiota was more similar within individuals over time than between subjects, with the fecal microbiota being unique from that of the small intestine. The stomach and duodenal microbiota displayed highest intraindividual variability compared to jejunal sites, which were more stable across time. We observed significant correlations in the duodenal microbial composition with changes in pH; linear mixed models identified positive correlations with multiple Streptococcus operational taxonomic units (OTUs) and negative correlations with multiple Prevotella and Pasteurellaceae OTUs. Few OTUs correlated with mesalamine concentration. The stomach and duodenal microbiota exhibited greater compositional dynamics than the jejunum. Short-term fluctuations in the duodenal microbiota were correlated with pH. Given the unique characteristics and dynamics of the proximal GI tract microbiota, it is important to consider these local environments in health and disease states.

**IMPORTANCE** The gut microbiota are linked to a variety of gastrointestinal diseases, including inflammatory bowel disease. Despite this importance, microbiota dynamics in the upper gastrointestinal tract are understudied. Our article seeks to understand what factors impact microbiota dynamics in the healthy human upper gut. We found that the upper gastrointestinal tract contains consistently prevalent bacterial OTUs that dominate the overall community. Microbiota variability is highest in the stomach and duodenum and correlates with pH.

## Abstract (precis)

The gut microbiota are linked to a variety of gastrointestinal diseases, including inflammatory bowel disease. Despite this importance, microbiota dynamics in the upper gastrointestinal tract are understudied. Our article seeks to understand what factors impact microbiota dynamics in the healthy human upper gut. We found that the upper gastrointestinal tract contains consistently prevalent bacterial OTUs that dominate the overall community. Microbiota variability is highest in the stomach and duodenum and correlates with pH.

## INTRODUCTION

The microbiota of the proximal gastrointestinal tract in humans represent an understudied yet highly relevant microbial community (1). Physiological processes such as gastric emptying, bile acid secretion, and the transit of food can influence the proximal gastrointestinal (GI) tract and disease development (2–5). However, there is limited information on how the microbiota in this region are related to these processes and how these impact health and disease throughout the GI tract.

Much of our knowledge about the involvement of the human GI microbiota in maintaining health and preventing disease has relied on fecal sampling, a noninvasive sampling method that is largely representative of the large intestine (6, 7). Although it is known that the microbiota across the GI tract vary in composition and density (8–10), studying the microbiota at these sites is difficult, limiting our knowledge to invasive procedures, specific patient populations, or single time points (1). Analyses of mucosal samples from autopsies, endoscopies, and colonoscopies have revealed that streptococci and lactobacilli—both members of the oral and esophageal microbiota—are abundant members of the jejunal and ileal microbiota (11–17). Studies using naso-ileal catheters and ileostoma effluent, which allow collection over time, have supported these conclusions and revealed that the small intestinal microbiota is highly dynamic over short time courses, likely reflective of physiological processes at the stomach-small intestine interface (18–21).

Understanding how the microbiota along the GI tract are related is of physiological relevance, particularly in relation to intestinal homeostasis and disease. Recent evidence suggests that the drug mesalamine, designed to reach high concentrations in the GI tract as a treatment for inflammatory bowel disease (IBD), may directly target the microbiota in addition to host effectors (22, 23). Interestingly, mesalamine is less effective in treating IBD in the upper GI tract, which manifests as Crohn’s disease, than the lower GI tract, which manifests as ulcerative colitis. It is possible that some of the effectiveness of mesalamine as a treatment for IBD, or lack thereof, is mediated by the microbiota, potentiating the need to characterize these microbial communities to a fuller extent in the context of mesalamine administration.

This study investigated the bacterial composition across the intact upper GI tract in the same healthy, fasted adults over time. We used a multilumen tube designed to sample multiple sites along the upper GI tract. As part of a previously published study aimed at measuring mesalamine dissolution, subjects were given a dose of mesalamine and the proximal GI tract lumen was sampled over time (24). We used these samples to (i) characterize and compare microbial community dynamics over time at multiple upper GI sites within an individual and (ii) identify how environmental factors, such as pH and the acute effect of mesalamine, shaped the microbiota. To the best of our knowledge, this is the first study to characterize the luminal microbiota across multiple upper GI sites over time within the same individual.

## RESULTS

### Study population.

Using a multichannel catheter with multiple aspiration points, samples collected from the upper GI tract of 8 healthy subjects during 10 different study visits were processed for 16S microbial community analysis (24) (Table 1; see Text S1 and Table S1 in the supplemental material). Samples were collected hourly up to 7 h primarily from the proximal GI tract in the following possible locations: the stomach (*n* = 44), duodenum (*n* = 64), proximal/mid/distal jejunum (*n* = 46), and stool (*n* = 3). At the beginning of the study, subjects were given one form of mesalamine (Table 1). One of the seven subjects was studied three times over the course of 10 months; for most analyses, each study visit from this subject was considered independently.

**TABLE 1:** Subject recruitment

Subject^*a*^	Mesalamine formulation^*b*^	Age (yr)	BMI	Sex	No. of samples from:						
					Stomach	Duodenum	Jejunum			Stool	Total
							Proximal	Mid	Distal		
M046-A	Pentasa	38	21.2	M	1	8		7		1	17
M046-B	Apriso	38	21.3	M		8		5	6		19
M046-C	Lialda	38	21.7	M	8	6		7		1	22
M047	Pentasa	36	21.1	M		8	6				14
M048	Apriso	51	34.3	F	5	7					12
M053	Apriso	34	25.2	F	1		7	3			11
M061	Pentasa	51	21.6	M	7	8					15
M062	Pentasa	37	27.3	M	7	7				1	15
M063	Lialda	26	28.6	M	7	5		5			17
M064	Lialda	25	27.5	F	8	7					15
Summary	40% Pentasa, 30% Apriso, 30% Lialda	37 ± 8.6	25 ± 4.4	70% M	44	64	13	27	6	3	157

a Shown are selected metadata and sample collection demographics for 10 admissions. Subject M046 was admitted for three visits: A, B, and C. All subjects identified as Caucasian, and none identified as Hispanic/Latinx.

b Pentasa has immediate release in stomach acid, Apriso has extended release at a pH of >6, and Lialda has extended release at a pH of >7.

10.1128/mSphere.00126-19.1 TEXT S1 Description in added detail of how the catheter tube was sterilized and how DNA was prepared for Illumina MiSeq sequencing. Download Text S1, PDF file, 0.2 MB. Copyright © 2019 Seekatz et al. 2019 Seekatz et al. This content is distributed under the terms of the Creative Commons Attribution 4.0 International license.

10.1128/mSphere.00126-19.5 TABLE S1 Subject metadata. Shown are selected metadata and the relative abundance of operational taxonomic units (OTUs) for each sample used in the present study. Download Table S1, XLSX file, 0.4 MB. Copyright © 2019 Seekatz et al. 2019 Seekatz et al. This content is distributed under the terms of the Creative Commons Attribution 4.0 International license.

### The proximal GI microbiota are dominated by *Firmicutes* and distinct from the fecal microbiota.

**FIG 1:**
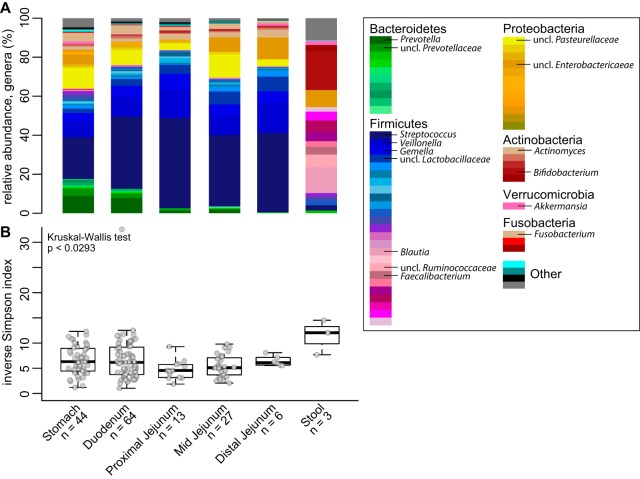
Bacterial community relative abundance and diversity in the upper GI tract. (A) The mean relative abundance of genera at each GI site (sample number [*n*] is indicated). (B) Box plots of the inverse Simpson index measuring community diversity across the GI tract (shown as median with first and third interquartile ranges). Statistical analysis was performed with the Kruskal-Wallis test (not significant).

Analysis of the relative abundances of 16S rRNA-encoding genes from the GI tract across all time points and individuals demonstrated that the small-intestinal microbiota was compositionally unique compared to stool (Fig. 1A). At all four sites in the proximal GI tract, Firmicutes composed the most abundant phyla (i.e., Streptococcus, Veillonella, and Gemella). Higher levels of Bacteroidetes species (Prevotella) were detected in the stomach and duodenum. Proteobacteria and Actinobacteria predominated in the remainder of the community at all sites. Diversity of the microbiota (inverse Simpson index) was decreased in sites of the upper GI tract compared to stool, which was enriched in *Firmicutes* (Blautia, Ruminococcaceae, and Faecalibacterium) and depleted in *Bacteroidetes* in these individuals (*n* = 3) (Fig. 1B).

### The proximal GI microbiota are individualized and variable over time.

**FIG 2:**
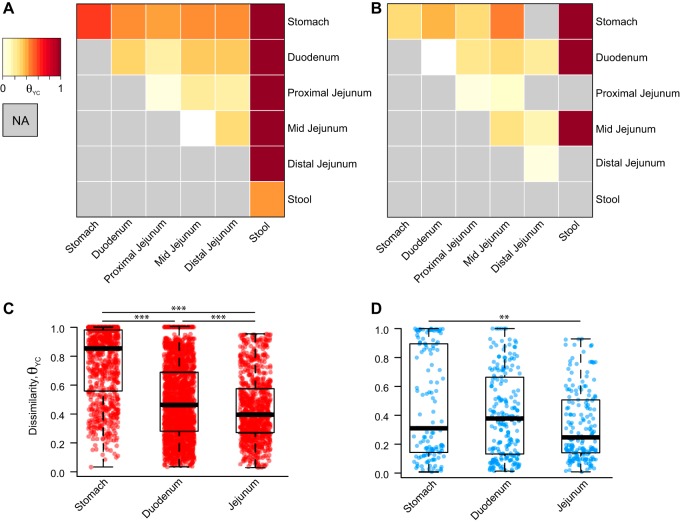
Dissimilarity of the proximal GI tract within and across individuals. (A and B) Heat map of the Yue and Clayton dissimilarity index (θ_YC_), comparing different proximal GI sites and stool across individuals (interindividual pairwise comparisons) (A) and within individuals (intraindividual pairwise comparisons) (B). (C and D) Interindividual (C) and intraindividual (D) dissimilarities in the stomach, duodenum, and jejunum (sites combined). Statistical analysis was performed with the Kruskal-Wallis test. We plot each sample at a given site rather than site averages, since this allows us to capture potential extreme states that those communities might adopt over time. Statistical analyses were performed with Dunn’s test for multiple comparisons with a Benjamini-Hochberg *P* value adjustment: **, *P* < 0.001; ***, *P* < 0.0001.

To compare the microbiota across the proximal GI tract within and across individuals, we assessed pairwise community dissimilarity using the Yue and Clayton dissimilarity index (θ_YC_), which takes into account relative abundance of operational taxonomic unit (OTU) compositional data. Both across (interindividual) and within (intraindividual) subjects, stool was highly dissimilar to any proximal GI site (Fig. 2A and B). Across proximal GI sites, subjects were more similar to their own samples than samples across other individuals (Fig. 2A to D). The stomach microbiota were highly dissimilar across individuals compared to the duodenum or any part of the jejunum, which exhibited the least amount of dissimilarity (Fig. 2C). A similar degree of dissimilarity was observed within an individual in the stomach, duodenum, and combined parts of the jejunum (Fig. 2D).

**FIG 3:**
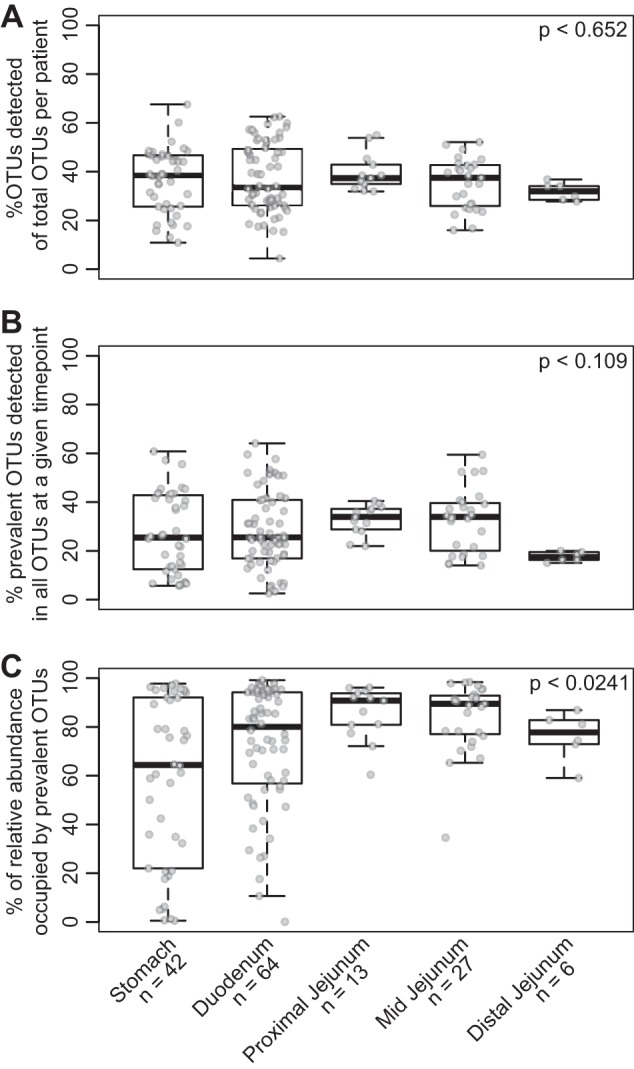
Fluctuations in prevalent OTUs observed within an individual across the proximal GI tract. (A) Box plots of the percentage of OTUs detected in a given sample out of all OTUs detected (all OTUs possible for that individual) at a subject site. (B) Box plots of the percentage of OTUs that were consistently detected at a subject site out of the total OTUs detected in a given sample. (C) The percentage of relative abundance explained by prevalent OTUs at a subject site in a given sample. Statistical analyses were performed with the Kruskal-Wallis test.

Using a dissimilarity measure such as θ_YC_ allowed us to assess stability based on changes in the relative abundance of OTUs. It is possible that certain GI sites fluctuate more in total OTUs. To measure whether any site had a higher rate of flux in their community (i.e., a higher rate of OTU turnover), we calculated the percentage of OTUs detected at a given time point from the total number of OTUs detected within that individual at a given site. We observed that for each proximal GI site, a mean of 36.6% of the OTUs ever detected in that subject at a given site (mean number of total OTUs ever detected per subject per site = 135; range, 78 to 212) were detectable at a given time point (Fig. 3A). Similarly, we calculated the number of OTUs that were consistently present in all samples collected at that site within an individual (mean number of consistently detected OTUs per subject per site = 14.1; range, 2 to 45). Overall, only 28.7% of the total OTUs ever detected at a given time point within an individual at a given site were represented by these consistently prevalent OTUs (Fig. 3B). However, these prevalent OTUs explained an average of 72.0% of the relative abundance observed in the samples (Fig. 3C). Of all sites, the relative abundance explained by the individual’s most prevalent OTUs in the stomach was lowest, followed by the duodenum, suggesting more variation at these sites compared to the jejunum (Kruskal-Wallis, *P* < 0.05).

One subject (M046) returned three times over the course of 10 months, allowing us to compare long-term changes. Across the sites that were sampled during multiple visits (the duodenum and mid-jejunum), prevalent OTUs were still detected during all three visits, explaining 74.4% and 66.1% OTUs in the duodenum and mid-jejunum, respectively (see Fig. S1 in the supplemental material).

10.1128/mSphere.00126-19.2 FIG S1 Fluctuations in prevalent OTUs observed within subject M046 across the proximal GI tract over the course of three visits. Shown are box plots of (A) the percentage of OTUs detected in a given sample out of all OTUs detected (all OTUs possible for that individual), (B) the percentage of OTUs that were consistently detected at a subject site out of the total OTUs detected in a given sample at a subject site, and (C) the percentage of relative abundance explained by prevalent OTUs at a subject site in the duodenum or stomach. The left side of the panel shows the data when the subject is treated as three separate admissions, and the right side shows the data when the subject is treated as the same individual across the board. (For example, a prevalent OTU would have to be present in all duodenal samples across all three visits to be considered a prevalent OTU in panel B.) Statistical analyses were performed with the Kruskal-Wallis test. Download FIG S1, PDF file, 0.07 MB. Copyright © 2019 Seekatz et al. 2019 Seekatz et al. This content is distributed under the terms of the Creative Commons Attribution 4.0 International license.

### Large fluctuations in the duodenal microbiota are associated with pH but not mesalamine.

**FIG 4:**
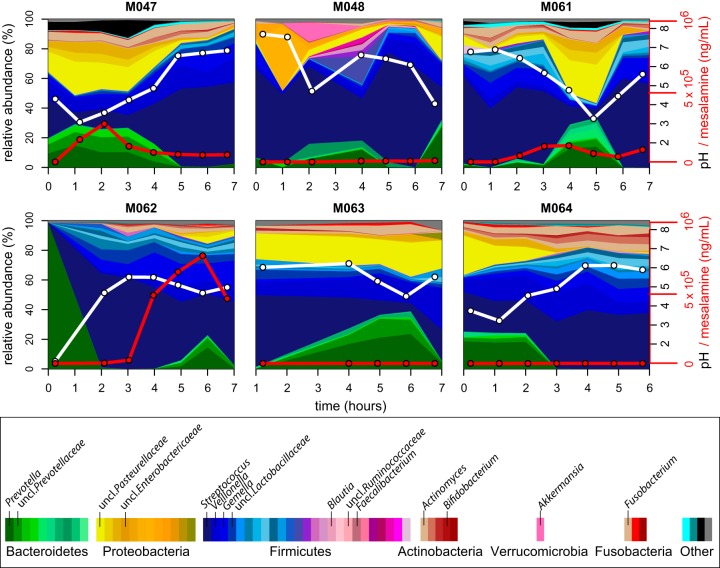
Longitudinal compositional dynamics, mesalamine levels, and pH in the duodenum. Shown are stream plots of genus-level composition over time in the duodenum of six individuals (percentage as indicated on the left *y* axis, with genera color-coded according to the color key at the bottom). White lines indicate pH measurements (black *y*-axis labels on the right), and red lines indicate mesalamine concentration (red *y-*axis labels on the right).

We next investigated how these compositional trends changed over time across the subjects. We focused on the duodenum and stomach since these sites were highly sampled across and within individuals and demonstrated variable pH. In the duodenum, we observed large fluctuations in genus-level composition across hourly time points within individuals (Fig. 4; see Fig. S2 and S3 in the supplemental material). Specifically, the relative abundance of *Streptococcus*, *Prevotella*, and an unclassified Pasteurellaceae species fluctuated in all individuals. We hypothesized that these fluctuations could be driven by mesalamine, administered in different forms to each subject at study onset. However, no visible pattern was observed with mesalamine levels. Interestingly, we observed that these compositional changes tracked with pH fluctuations (Fig. 4). These patterns were less apparent in the stomach, where individuals displayed variable dynamics and highly individualized compositional patterns independent of mesalamine levels or pH. A similar trend was observed in the jejunum of the subject with three different admissions, where pH fluctuated less (Fig. S1 and S2).

10.1128/mSphere.00126-19.3 FIG S2 Longitudinal compositional dynamics, mesalamine levels, and pH in the stomach. Shown are stream plots of genus-level composition over time in the stomach of six individuals (percentage as indicated on the left *y* axis, with the genera color-coded according to the key at the bottom). White lines indicate pH measurements (black *y*-axis labels on the right), and red lines indicate mesalamine concentration (red *y*-axis labels on the right). Download FIG S2, PDF file, 0.4 MB. Copyright © 2019 Seekatz et al. 2019 Seekatz et al. This content is distributed under the terms of the Creative Commons Attribution 4.0 International license.

10.1128/mSphere.00126-19.4 FIG S3 Longitudinal compositional dynamics, mesalamine levels, and pH in the duodenum and jejunum of subject M046. Shown are stream plots of genus-level composition over time in the duodenum (upper panels) and jejunum (lower panels) of one individual across three different visits (percentage as indicated on the left *y* axis, with genera color-coded in the key at the bottom). White lines indicate pH measurements (black *y*-axis labels on right), and red lines indicate mesalamine concentration (red *y*-axis labels on right). Download FIG S3, PDF file, 0.3 MB. Copyright © 2019 Seekatz et al. 2019 Seekatz et al. This content is distributed under the terms of the Creative Commons Attribution 4.0 International license.

**FIG 5:**
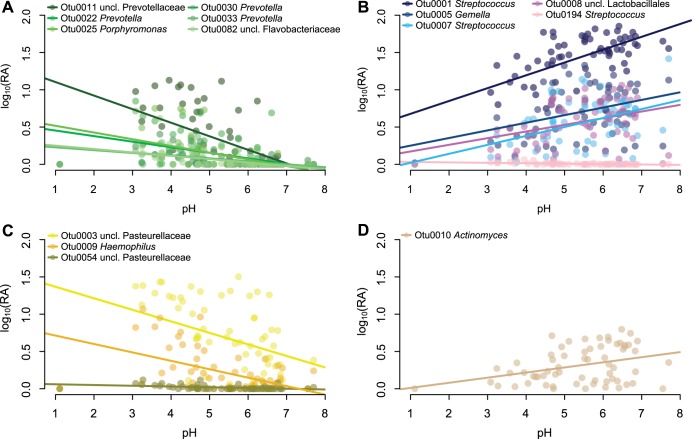
Relative abundance of significant OTUs versus pH. Shown is the log relative abundance [log_10_(RA)] as a function of pH of OTUs found to be significantly correlated with pH using linear mixed models (all samples with measurable pH). Lines represent linear fit per OTU. OTUs classified as *Firmicutes* (A), *Bacteroidetes* (B), *Proteobacteria* (C), and *Actinobacteria* (D) are depicted. The genus-level OTU classification is defined in the color code key in each panel.

To identify whether any singular OTUs correlated with changes in pH, we applied a generalized linear mixed-model approach that takes into account subject specificity (25–27). Within duodenal samples (*n* = 56), we observed 15 OTUs that significantly correlated with pH changes. Linear regression of pH and relative abundance of these OTUs were significant across all samples (Fig. 5; see Table S2 in the supplemental material). Of the negatively correlated OTUs, six OTUs were classified as *Bacteroidetes*—mainly *Prevotella*—and two OTUs were classified as *Pasteurellaceae* (*Proteobacteria*). The majority of the OTUs that were positively correlated with pH were *Firmicutes*, mainly *Streptococcus*, alongside an Actinomyces OTU (*Actinobacteria*). Only one OTU in the duodenum was significantly correlated to mesalamine (Table S2). We identified 17 OTUs that correlated with pH or mesalamine in the stomach; however, these were not representative at all sites (Table S2).

10.1128/mSphere.00126-19.6 TABLE S2 Generalized linear mixed-modeling results. Shown are OTUs that were significantly correlated with pH or mesalamine in the stomach or duodenum. Download Table S2, XLSX file, 0.05 MB. Copyright © 2019 Seekatz et al. 2019 Seekatz et al. This content is distributed under the terms of the Creative Commons Attribution 4.0 International license.

## DISCUSSION

Our results demonstrate that the microbial communities inhabiting the GI tract are distinct and dynamic across different sites within the proximal GI tract. Our sampling procedure provided us with an opportunity to longitudinally characterize such microbial populations in conjunction with the administration of a commonly used drug, mesalamine. We observed high stability of the microbiota in the jejunum compared to the stomach or duodenum, indicating that the indigenous microbiota residing in more proximal regions of the GI tract may experience greater changes. While we did not observe strong correlations between mesalamine concentration and particular microbiota members at any site, we did observe a strong correlation between the microbiota composition and pH, particularly in the duodenum.

In this report, we describe the use of a multilumen catheter design with unique aspiration ports that enabled sampling of small-intestinal content over the course of 7 h (24). Many studies aimed at investigating the microbiota of the proximal GI have overcome sampling difficulty in this region by using ileostoma effluent, samples from newly deceased individuals, or naso-ileal tubes. Although easy to access, ileostoma effluent does not fully recapitulate the distal small intestine, as it more closely resembles the colon than the small intestine due to increased oxygen concentrations near the stoma (28–31). Single lumen naso-ileal tubes are unable to sample multiple sites simultaneously (18, 20, 21, 32). GI fluid collected with our methodology was sufficient for determining mesalamine concentration, assaying fluid pH, and isolating microbial DNA across time and GI sites, which has not been previously described (24).

Our results support previous observations that the small intestine is dynamic with higher interindividual than intraindividual variability (18, 21, 33). However, the mid- to distal small intestine also contains a resilient microbial community composed of several highly abundant OTUs. This resilience is demonstrated by the shift from an altered to a normal ileal microbiota following the resolution of an ileostoma (34). This mirrors the colonic microbiota, which also has a small community that is stable over long periods of time (30, 35, 36).

This and other studies have shown that the jejunal and proximal ileal microbiota are distinct from the colonic microbiota (10, 37). Despite changes in overall community structure and an overall decrease in microbial diversity across the stomach and small intestine compared to stool, many of the same organisms commonly observed in stool were also present in the upper GI tract, albeit at very different abundances (10). Interestingly, colonic resection and ileal pouch-anal anastomosis have been shown to shift the terminal ileum microbiota to a state similar to the colon, suggesting that a colonic community structure can develop at these sites given the right conditions (21, 31, 37–39).

Many of the abundant microbes observed in our study (*Streptococcus*, *Veillonella*, *Gemella*, and *Pasteurellaceae* species) are also common residents of the oral cavity, which reflects the proximity of these locations in the GI tract. Populations of *Proteobacteria*, such as *Pasteurellaceae*, have also been observed consistently in the small-intestinal microbiota in other studies, particularly in patients with IBD (14, 40–42). In our study, *Streptococcus* and *Veillonella* were correlated with pH in duodenal samples. It is possible that growth of these organisms drives a decrease in pH via metabolism of short-chain fatty acids, an observed functional capacity of these genera (21, 43). Conversely, large fluctuations in environmental pH may select for genera like *Streptococcus*, which have evolved a variety of mechanisms to control pH intracellularly (44–47). In any case, our data suggest a relationship between microbial dynamics and environmental physiology of the duodenum, which is an important observation to consider when comparing this site across individuals.

We observed little association between mesalamine concentration and changes in microbial relative abundance in our cohort. Several studies have reported differences in the fecal microbiota of patients with or without IBD, in particular Crohn’s disease, which can affect the small intestine (40). Compositional shifts in the small intestine have been reported during IBD, specifically increased levels of Enterobacteriaceae species, such as Enterococcus, Fusobacterium, or Haemophilus (14, 41, 42). It has been hypothesized that mesalamine’s ability to reduce inflammation in patients with ulcerative colitis could be by altering the microbiota (22, 23). While acute effects of mesalamine on the microbiota have not previously been reported, earlier work has demonstrated that mesalamine decreases bacterial polyphosphate accumulation and pathogen fitness, suggesting an influence on the microbiota (23). We did not observe strong correlations between mesalamine concentration and the microbiota here. However, our study was small, used different doses of mesalamine that may be metabolized differently across GI sites, and was conducted in healthy individuals (24). It is possible that mesalamine is less likely to impact the small-intestinal microbiota compared to the large intestine; indeed, mesalamine is historically known to have a lower efficacy in treating Crohn’s disease, which manifests in the small intestine, compared to ulcerative colitis, which manifests in the large intestine (22, 48, 49). As indicated by the variability of mesalamine in the subjects in this study, the effects of mesalamine on the small-intestinal microbiota may be highly individualized (24, 50–52). Furthermore, individuals with disease may harbor a distinct microbiota that responds to mesalamine differently.

Despite the opportunity provided by our method to describe the microbiota across the GI tract, our study has some lingering questions. Movement by the subject during the study can result in movement of each sampling port, particularly between the distal stomach and antrum. This may explain the inconsistent pH values and severe fluctuations of the microbiota observed in the stomach. Similarly, the shorter length of the sampling device, compared to a naso-ileal catheter, prevented reliable collection of fluid from the distal small intestine, limiting our sampling to the proximal region. We also were limited to three concurrent fecal samples, each of which was low in *Bacteroidetes*, a profile generally observed in individuals on low-fat, high-fiber, non-Western diets (53). While this could have been due to the influence of mesalamine on the colonic microbiota, we did not have a sufficient *n* to test this hypothesis.

The use of a novel catheter allowed us to assess the microbiota across several proximal GI sites over time, representing a powerful clinical and/or investigative tool for studying the small-intestinal microbiota. Future studies on the upper GI microbiota should collect concurrent oral swab/sputum and fecal samples to strengthen the ability to “track” microbial populations across the GI tract, potentiating our ability to correlate the microbiota from fecal sampling, a more convenient method to study the microbiota, to other sites of the GI tract.

## MATERIALS AND METHODS

### Study recruitment.

Healthy individuals (ages 18 to 55 years) were included who were free of medications for the past 2 weeks, passed a routine health screening, had a body mass index (BMI) of 18.5 to 35, and had no significant clinical illness within 3 weeks. Health screening included a review of medical history and a physical examination (checking vital signs, electrocardiography, and clinical laboratory tests) as described by Yu et al. (24).

### Catheter design and sterilization.

A customized multichannel catheter was constructed by Arndorfer, Inc. (Greendale, WI), consisting of independent aspiration ports located 50 cm apart. The catheter had a channel to fit a 0.035-in. by 450-cm guide wire (Boston Scientific, Marlborough, MA), a channel connected to a balloon that could be filled with 7 ml of water to assist tube placement, and an end that was weighted with 7.75 g of tungsten. Each single-use catheter was sterilized according to guidelines set by the American Society for Gastrointestinal Endoscopy at the University of Michigan prior to insertion (54) (Text S1).

### Collection of GI fluid samples.

The full details of catheter placement have been described previously (24). Briefly, catheter placement occurred approximately 12 h before sample collection. The catheter was orally inserted into the GI tract, with aspiration ports located in the stomach, duodenum, and the proximal, mid-, and distal jejunum, confirmed by fluoroscopy. Subjects were given a light liquid snack approximately 11 h before sample collection and fasted overnight for 10 h prior to sample collection. At 0 h, a mesalamine formulation was administered to each subject (Table 1). Luminal GI fluid samples (approximately 1.0 ml) were collected from up to four sites of the upper GI tract hourly up to 7 h. Samples were collected by syringe, transferred to sterile tubes, and placed at −80°C until sample processing. A paired sample was collected to detect pH using a calibrated micro-pH electrode (Orion pH probe catalog no. 9810BN; Thermo Scientific, Waltham, MA).

### DNA extraction and Illumina MiSeq sequencing.

The detailed protocol for DNA extraction and Illumina MiSeq sequencing was followed as previously described with modifications (55) (Text S1). Briefly, 0.2 ml of GI fluid or 20 mg of stool was used for DNA isolation using a Qiagen (Germantown, MD) MagAttract Powermag microbiome DNA isolation kit (catalog no. 27500-4-EP). Barcoded dual-index primers specific to the V4 region of the 16S rRNA gene were used to amplify the DNA (56), using a “touchdown PCR” protocol (Text S1). Multiple negative controls were run parallel to each PCR. PCR mixtures were normalized, pooled, and quantified (56). Libraries were prepared and sequenced using the 500-cycle MiSeq V2 reagent kit (catalog no. MS-102-2003; Illumina, San Diego, CA). Raw FASTQ files, including those for negative controls, were deposited in the Sequence Read Archive (SRA) database.

### Data processing and microbiota analysis.

Analysis of the V4 region of the 16S rRNA gene was done using mothur (v1.39.3) (56, 57). Full methods, including detailed processing steps, raw processed data, and code for each analysis, are described in GitHub. Briefly, following assembly, quality filtering, and trimming, reads were aligned to the SILVA 16S rRNA sequence database (v128) (58). Chimeric sequences were removed using UCHIME (59). Prior to analysis, both mock and negative-control samples (water) were assessed for potential contamination; samples with <2,500 sequences were excluded (Table S1). Sequences were binned into operational taxonomic units (OTUs), with 97% similarity, using the opticlust algorithm (60). The Ribosomal Database Project (v16) was used to classify OTUs or sequences directly for compositional analyses (>80% confidence score) (61). Alpha and beta diversity measures (inverse Simpson index and the Yue and Clayton dissimilarity index [θ_YC_]) were calculated from unfiltered OTU data (62). Basic R commands were used to visualize results, calculate percentage of OTUs shared between samples, and conduct statistics, using the packages plyr, dplyr, gplots, tidyr, and tidyverse. The nonparametric Kruskal-Wallis test, using Dunn’s test for multiple comparisons and adjusting *P* values with the Benjamini-Hochberg method when indicated, was used for multigroup comparisons. The R packages lme4 and lmerTest were used for mixed linear models for comparisons between OTU relative abundance (filtered to include OTUs present in at least half of samples collected from a subject per site) and pH or mesalamine (63, 64).

### Ethics approval and consent to participate.

Samples collected in this study were part of clinical trial NCT01999400. The institutional review boards at the University of Michigan (IRBMED) and the Department of Health and Human Services, Food and Drug Administration (Research Involving Human Subjects Committee [RIHSC]), both approved the study protocol (IRB approved on 4 February 2015). All subjects provided written informed consent in order to participate. Informed consent was obtained from individuals prior to the time of sampling.

### Availability of data.

Raw FASTQ files, including those for negative controls, were deposited in the SRA database under BioProject ID no. PRJNA495320 and BioSample ID no. SAMN10224451 to SAMN10224634. Detailed processing steps, raw processed data, and code for each analysis are described in GitHub at https://github.com/aseekatz/SI_mesalamine.
