# Supplementary material for: Spatial and Temporal Analysis of the Stomach and Small-Intestinal Microbiota in Fasted Healthy Humans
Source: mSphere. 2019 Mar 13;4(2):e00126-19. doi: 10.1128/mSphere.00126-19 (PMC6416366; doi:10.1128/mSphere.00126-19)
Supplement: TEXT S1 [file mSphere.00126-19-s0001.pdf]

SUPPLEMENTAL METHODS

Catheter tube sterilization

Sterilization of the single-use catheters were conducted according to guidelines set by the American Society for Gastrointestinal Endoscopy at the University of Michigan prior to insertion (1). Briefly, each tube was flushed with an enzymatic presoak detergent Asepti-Zyme (Ecolab, St. Paul, MN), let sit for two minutes, then flushed with water. Each tube was then flushed with MetriCide (Metrex, Orange, CA), containing the active ingredient Glutaraldehyde (2.6%). Each tube sat for 20 minutes in MetriCide then was rinsed with tap water and flushed with a 70% isopropanol solution, purging any remaining alcohol using forced air.

DNA extraction and Illumina MiSeq sequencing

The detailed protocol for DNA extraction and Illumina MiSeq sequencing was followed as previously described with modifications (Supplemental Methods) (2). Briefly, 0.2 ml of GI fluid or 20 mg of stool was used for DNA isolation using a Qiagen (Germantown, MD) MagAttract Powermag microbiome DNA isolation kit (catalog no. 27500-4-EP). Barcoded dual-index primers specific to the V4 region of the 16S rRNA gene were used to amplify the DNA (3). The PCR reactions included the following: 5 µl of 4 µM equimolar primer set, 0.15 µl of AccuPrime Taq DNA High Fidelity Polymerase, 2 µl of 10x AccuPrime PCR Buffer II (Thermo Fisher Scientific, Waltham, MA, catalog no. 12346094), 11.85 µl of PCR-grade water, and 1 µl of DNA template. A “touchdown PCR” protocol was performed using the following conditions: 2 min at 95°C, followed by 20 cycles of 95°C for 20 s, 60°C for 15 s, and

72°C for 5 min (with a 0.3°C decrease of the 60°C annealing temperature each cycle), followed by 20 cycles of 95°C for 20 s, 55°C for 15 s, and 72°C for 5 min, followed by 72°C for 10 min. Multiple water samples (negative controls) were ran parallel to each PCR reaction. Each PCR reaction was normalized using a SequalPrep Normalization Plate Kit (Thermo Fisher Scientific, catalog no. A1051001). The normalized reactions (normalized to the lowest concentration of the pooled plates) were pooled and quantified using the Kapa Biosystems (Wilmington, MA) Library qPCR MasterMix (ROX Low) Quantification kit for Illumina platforms (catalog no. KK4873). The Agilent Bioanalyzer was used to confirm the size of the amplicon library (~399 bp) using a high-sensitive DNA analysis kit (Agilent, Santa Clara, CA, catalog no. 5067-4626). The pooled amplicon library was sequenced on the Illumina MiSeq platform using the 500 cycle MiSeq V2 Reagent kit (Illumina, San Diego, CA, catalog no. MS-102-2003) according to the manufacturer's instructions with modifications of the primer set with custom read 1/read 2 and index primers added to the reagent cartridge. Libraries were prepared according to “Preparing Libraries for Sequencing on the MiSeq” (part 15039740, Rev. D) protocol. The final load concentration was 5.5 pM with a 15% PhiX spike to add diversity within the run.

1. Committee AQAIE, Petersen BT, Chennat J, Cohen J, Cotton PB, Greenwald DA, Kowalski TE, Krinsky ML, Park WG, Pike IM, Romagnuolo J, Society for Healthcare Epidemiology of A, Rutala WA. 2011. Multisociety guideline on reprocessing flexible gastrointestinal endoscopes: 2011. Gastrointest Endosc 73:1075-84.
2. Seekatz AM, Theriot CM, Molloy CT, Wozniak KL, Bergin IL, Young VB. 2015. Fecal Microbiota Transplantation Eliminates Clostridium difficile in a Murine Model of Relapsing Disease. Infect Immun 83:3838-46.
3. Kozich JJ, Westcott SL, Baxter NT, Highlander SK, Schloss PD. 2013. Development of a dual-index sequencing strategy and curation pipeline for analyzing amplicon sequence data on the MiSeq Illumina sequencing platform. Appl Environ Microbiol 79:5112-20.
