# Supplementary figures and images for: Spatial and Temporal Analysis of the Stomach and Small-Intestinal Microbiota in Fasted Healthy Humans
Source: mSphere. 2019 Mar 13;4(2):e00126-19. doi: 10.1128/mSphere.00126-19 (PMC6416366; doi:10.1128/mSphere.00126-19)

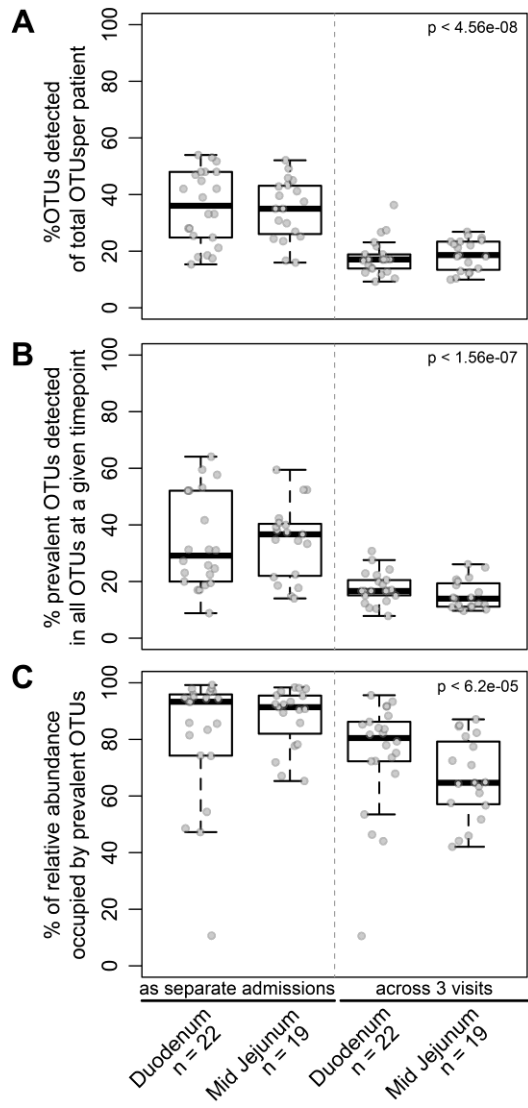

Supplement: FIG S1 [file mSphere.00126-19-sf001.pdf]

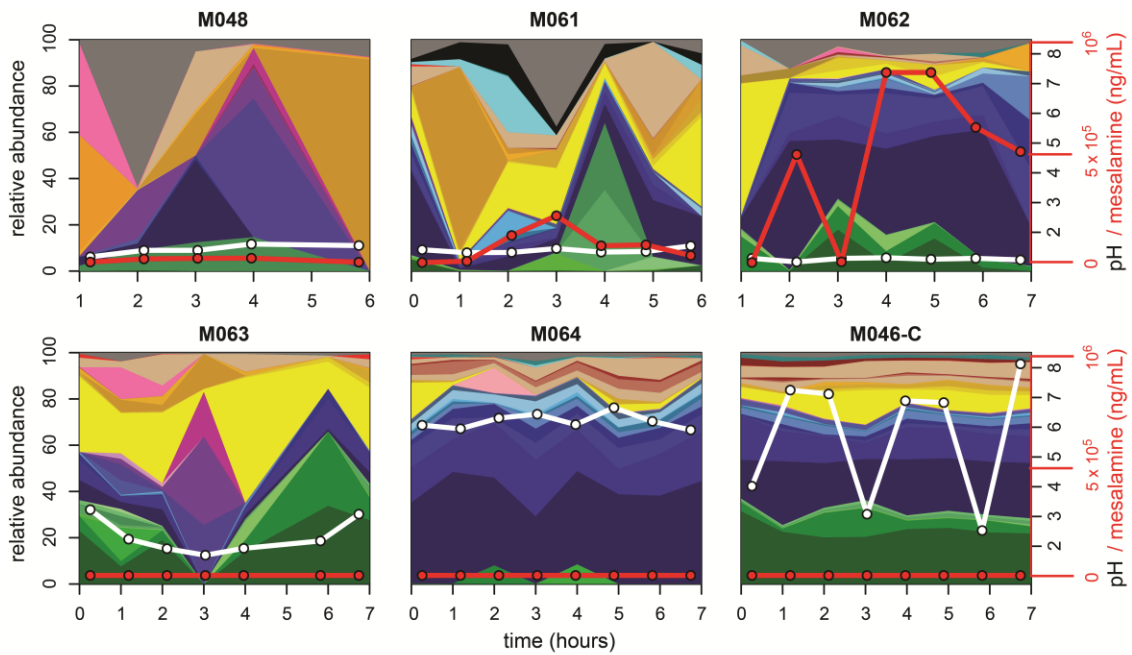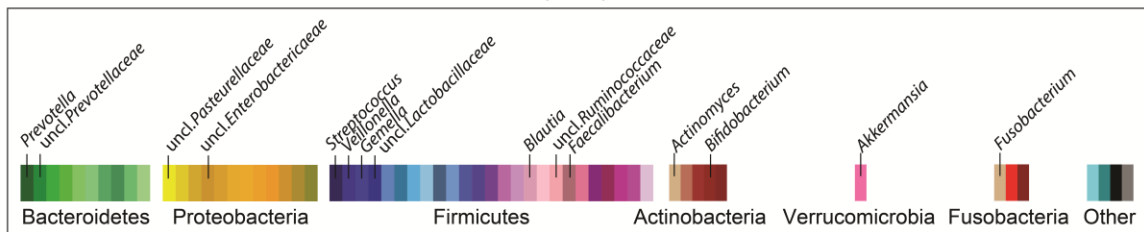

Supplement: FIG S2 [file mSphere.00126-19-sf002.pdf]

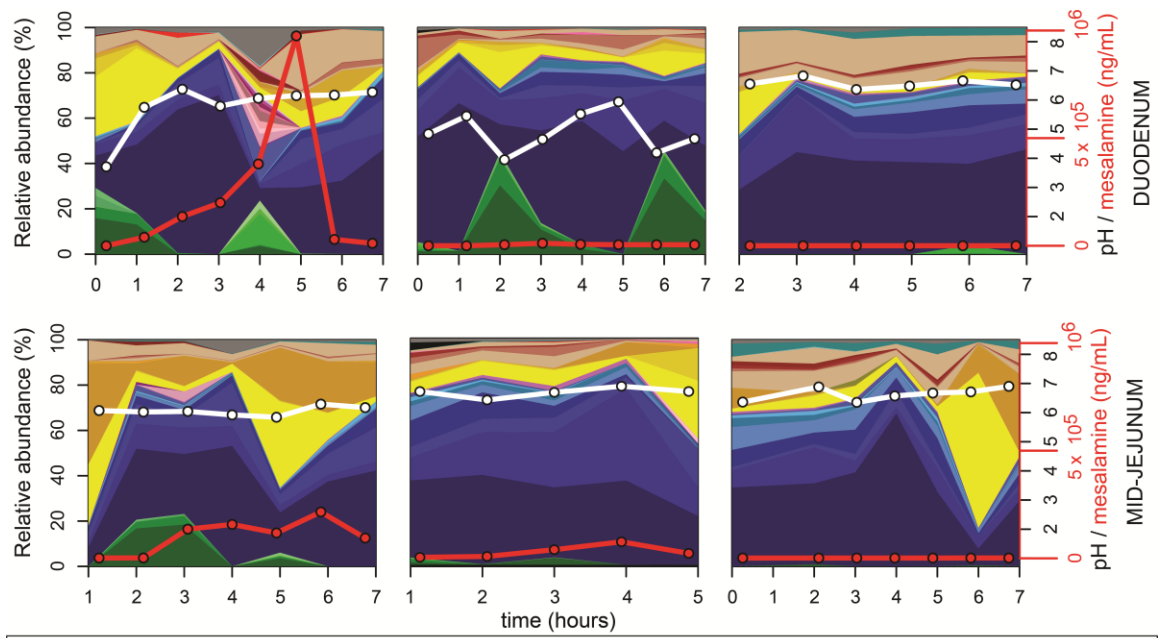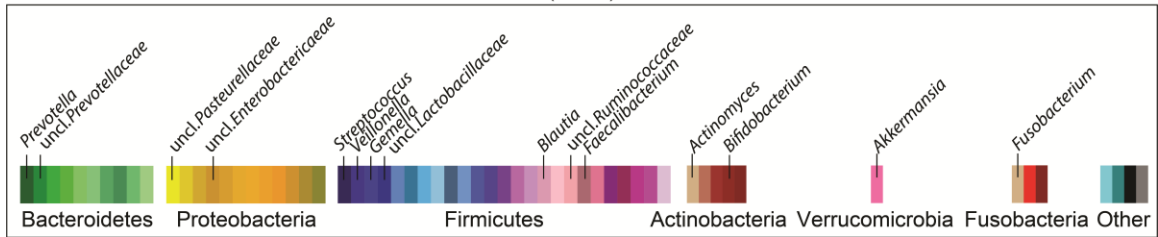

Supplement: FIG S3 [file mSphere.00126-19-sf003.pdf]
